# Effect of Family Income on the Relationship Between Parental Education and Sealant Prevalence, National Health and Nutrition Examination Survey, 2005–2010

**DOI:** 10.5888/pcd12.150037

**Published:** 2015-08-27

**Authors:** Dania E. Al Agili, Susan O. Griffin

**Affiliations:** Author Affiliations: Dania E. Al Agili, King Abdulaziz University, Jeddah, Saudi Arabia.

## Abstract

**Introduction:**

We examined the association between sealant prevalence and parental education for different levels of family income, controlling for other covariates.

**Methods:**

We combined data from 2005–2006, 2007–2008, and 2009–2010 cycles of the National Health and Nutrition Examination Survey. The study sample was 7,090 participants aged 6 to 19 years. Explanatory variables, chosen on the basis of Andersen and Aday’s framework of health care utilization, were predisposing variables — child’s age, sex, race/ethnicity, and parental education (<high school diploma; high school diploma; >high school diploma); enabling variables — family income (<100% of the federal poverty level [FPL]; 100%–200% of the FPL; and >200% of the FPL), health insurance status, and regular source of medical care; and a need variable — future need for care (perceived child health status is excellent/very good, good, fair/poor). We conducted bivariate and multivariate analyses and included a term for interaction between education and income in the multivariate model. We report significant findings (*P* ≤ .05).

**Results:**

Sealant prevalence was associated with all explanatory variables in bivariate and multivariate analyses. In bivariate analyses, higher parental education and family income were independently associated with higher sealant prevalence. In the multivariate analysis, higher parental education was associated with sealant prevalence among higher income children, but not among low-income children (<100% FPL). Sealant prevalence was higher among children with parental education greater than a high school diploma versus less than a high school diploma in families with income ≥100% FPL.

**Conclusion:**

Our findings suggest that income modifies the association of parental education on sealant prevalence. Recognition of this relationship may be important for health promotion efforts.

## MEDSCAPE CME

Medscape, LLC is pleased to provide online continuing medical education (CME) for this journal article, allowing clinicians the opportunity to earn CME credit.

This activity has been planned and implemented in accordance with the Essential Areas and policies of the Accreditation Council for Continuing Medical Education through the joint sponsorship of Medscape, LLC and *Preventing Chronic Disease*. Medscape, LLC is accredited by the ACCME to provide continuing medical education for physicians.

Medscape, LLC designates this Journal-based CME activity for a maximum of 1 ***AMA PRA Category 1 Credit(s)™***. Physicians should claim only the credit commensurate with the extent of their participation in the activity.

All other clinicians completing this activity will be issued a certificate of participation. To participate in this journal CME activity: (1) review the learning objectives and author disclosures; (2) study the education content; (3) take the post-test with a 75% minimum passing score and complete the evaluation at www.medscape.org/journal/pcd; (4) view/print certificate.

**Release date: August 27, 2015; Expiration date: August 27, 2016**

### Learning Objectives

Upon completion of this activity, participants will be able to:

1. Evaluate the epidemiology of dental caries and the effectiveness of dental sealants
2. Assess how family income and educational attainment may affect the application of dental sealants
3. Distinguish the interaction between family income and educational attainment in the application of dental sealants
4. Identify other risk factors for the failure to apply dental sealants

**EDITORS**

Teresa Ramsey, Editor, *Preventing Chronic Disease*. Disclosure: Teresa Ramsey has disclosed no relevant financial relationships.

**CME AUTHOR**

Charles P. Vega, MD, Clinical Professor of Family Medicine, University of California, Irvine

Disclosure: Charles P. Vega, MD, has disclosed the following relevant financial relationships: Served as an advisor or consultant for: Lundbeck, Inc; McNeil Pharmaceuticals; Takeda Pharmaceuticals North America, Inc.

**AUTHORS AND CREDENTIALS**

Dania E. Al Agili, BDS, MS, MPH, DrPH Associate Professor, King Abdulaziz University, Jeddah, Saudi Arabia

 Disclosure: Dania E. Al Agili, BDS, MS, MPH, DrPH, has disclosed no relevant financial relationships.

Susan O. Griffin, PhD Health Economist, Division of Oral Health, Centers for Disease Control and Prevention, Atlanta, Georgia, USA

Disclosure: Susan O. Griffin, PhD, has disclosed no relevant financial relationships.

## Introduction

Despite marked improvements in the oral health of children and youth in the United States over the past decades, dental caries remains one of the most common chronic childhood diseases (1). By age 17, almost 70% of adolescents have experienced caries (2) and most (90%) caries in permanent teeth occurs in the pits and fissures (3). Dental caries disproportionately affect low-income children — 66% of adolescents living in poverty have experienced caries compared with 54% of children living in families with incomes greater than 200% of the federal poverty level (2). Although US children living in poverty are at higher risk for caries, only 1 in 4 has had at least 1 dental sealant (4).

Sealants are effective in preventing and controlling dental caries in the pits and fissures of permanent teeth (1). A recently published Cochrane review found that sealants reduced caries by 81% at 2-year follow-up (5). Another review found that placing sealants on noncavitated lesions reduced the progression of caries by 70% up to 5 years after placement (6). Increasing sealant prevalence among children at risk for caries is a national health objective (7). Both the Centers for Medicare and Medicaid Services (8) and the National Quality Forum (9) have endorsed performance measures related to increased sealant prevalence among Medicaid-enrolled and privately insured children, respectively, who are at risk for caries.

Factors associated with a child’s not having sealants include ability to pay for dental care (ie, low family income) (4); not having dental or health insurance (4); sociodemographic variables, including having parents who did not graduate high school (4); being of minority race/ethnicity (4); and low health literacy and low oral health literacy (10). A recent analysis found that parents’ functional health literacy and English being spoken at home were strong predictors of sealant prevalence among California school children (10). The RAND Health Insurance Experiment (HIE) also found that health literacy (ie, parents’ knowledge of the medical care system) predicted use of dental health services among children (11). Studies further suggest that one of the strongest predictors of medical and oral health literacy is educational attainment (12,13). Knowledge of the preventive benefits of sealants is also almost 5 times higher among people with more than a high school education compared with those without a high school education (34% vs 7%) (14).

Studies also indicate that factors affecting ability to pay for services may modify the effect of education on use of dental health services, although the direction of the effect varies by study. An earlier analysis of National Health Interview Survey (NHIS) data found that higher income predicted having a sealant only among children of parents who had a high school education or more (15). This analysis, however, was conducted in 1989 and reported that sealant prevalence among children aged 6 to 17 years was 16% (15), about half the current prevalence of 31% (4). Another early analysis from the RAND HIE found, however, that the presence of enabling resources (ie, removal of cost sharing from dental insurance plans) among less educated people resulted in more fillings and less untreated decay (16).

The predictive power of these factors largely can be explained in the context of Andersen and Aday’s predisposing, enabling, and need (PEN) model of health services’ use (17,18). According to the PEN model, health care utilization is a function of 3 factors: 1) demographic and social characteristics that influence a person’s attitudes and valuation of health, which in turn predispose a person to use care; 2) location and availability of health care as well as personal resources that enable a person to access care; and 3) a person’s perceived need for care. 

In this article, we examine the association between sealant prevalence and predisposing, enabling, and need variables. In light of the findings of the analysis of NHIS data, of special interest is whether the association between sealant prevalence and education, a predisposing variable, is still modified by income, an enabling resource. We hypothesize that predisposing variables will have higher predictive ability in the presence of sufficient enabling resources or alternatively that enabling resources will have higher predictive power among parents predisposed to use sealants.

## Methods

### Data source

The National Health and Nutrition Examination Survey (NHANES) is a cross-sectional survey conducted by the Centers for Disease Control and Prevention’s (CDC’s) National Center for Health Statistics (NCHS) to assess the health and nutritional status of the civilian, noninstitutionalized US population. (Additional information is available at www.cdc.gov/nchs/nhanes.htm.) The survey uses a complex, multistage probability sampling design. NHANES participants are interviewed in their homes and then complete a health examination at a mobile examination center. We used data from the NHANES interview questionnaire and the oral health examination combined for 2005–2006, 2007–2008, and 2009–2010. The response rate for the combined examined sample is 83.7%.

The NHANES oral health examination included a Basic Screening Examination (BSE) assessment where participants’ teeth were examined visually for presence of untreated dental caries, dental restorations, and dental sealants. In 2005–2006 and 2007–2008, the BSE was performed by health technologists among people aged 5 years and older; in 2009–2010, dental hygienists performed the BSE among participants aged 3 to 19 years only (19). The BSE in 2009–2010 made use of the same examination protocols that were used in 2005–2008. Comprehensive training and calibration of examiners are conducted throughout the continuous NHANES 2-year cycles. The kappa statistics for sealants between health technologists and the survey reference examiner ranged from 0.82 to 0.90 for 2005–2008 and the statistic between dental hygienists and the survey reference examiner was 0.71 in 2009–2010. These statistics could be directly compared, because the reference examiner did not change between the data collection periods (19,20).

**Figure 1:**
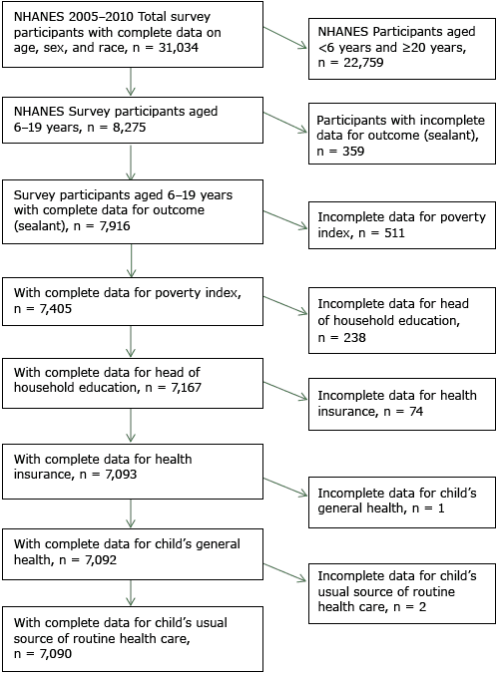
Selected study population of participants for substudy on prevalence of dental sealants among children and adolescents aged 6 to 19 years, National Health and Nutrition Examination Survey, 2005–2010.

We used data from the NHANES public-use files; therefore, CDC/NCHS Ethics Review Board approval was not needed. Of the 8,275 participants aged 6 to 19 years in NHANES, 7,916 had sealant data. Among these children, 749 children did not have data for family income, parental education, or both. Of the remaining 7,167 children, 74 did not have data on health insurance and 3 did not have data on general health status or usual source of care. The final study sample was 7,090 (Figure 1).

Dental sealant, the dependent variable, was recorded as present if at least 1 posterior primary or permanent tooth had a sealant, even if part of the sealant was not visible (2). Andersen and Aday’s PEN model was used for selecting independent variables (18). The main independent variable of interest was the predisposing factor, education level of head of household (referred to henceforth as “parental education”). Education was coded as “less than high school diploma,” “high school diploma,” or “more than high school diploma.” Other independent variables in this analysis included predisposing factors (child’s age, sex, and race/ethnicity), enabling factors (income, health insurance status, regular source of medical care), factors associated with future need for dental care (parent’s perceived health status of child), which studies suggest predicts oral health status of child (21), and survey year, to capture differences over time. Age was divided into 2 categories, 6 to 11 and 12 to 19 years, which coincide with eruption of the first and second permanent molars, around 6 and 12 years, respectively. The child’s race/ethnicity was coded as non-Hispanic white, non-Hispanic black, Mexican American, or other, which included other Hispanic, other races, or multiple races. The ratio of family income to federal poverty level (FPL) was categorized as less than 100% of the FPL, 100% to 200% of the FPL, or greater than 200% of the FPL. Four health insurance categories were created: no insurance; private or military; Medicaid or State Children’s Health Insurance Program (SCHIP); and other government health insurance (eg, state-sponsored health insurance, Indian health). Finally, parents’ perception of the child’s general health was coded as excellent or very good, good, or fair or poor.

SAS-Callable SUDAAN, which correctly estimates the variance for complex surveys, was used to generate estimates, standard errors (SEs), and associated confidence intervals (CIs) (SUDAAN software, release 11.0.0, RTI International). We used a χ^2^ test to determine if the characteristics of children in our study sample differed from those excluded from our study as well as to examine if sealant prevalence differed among our explanatory variables. All reported differences are significant at *P* ≤ .05 and all CIs are reported at the 95% level. We used logistic regression to identify predisposing, enabling, and need factors associated with having at least 1 sealant. To assess whether income modified the association between education and having sealants, our model included a term for interaction between these 2 variables. Point estimates of model-adjusted sealant prevalence for the 3 levels of parental education stratified by family income were also obtained from the average marginal predictions in the fitted logistic regression model (22). The model fit was assessed using the Hosmer-Lemeshow goodness-of-fit test.

## Results

Excluded children had less educated parents but lived in families with income levels similar to those of children included in this study (Table 1). Excluded children were also older, less likely to be non-Hispanic white, and more likely to be “other race/ethnicity.” Overall, among children aged 6 to 19 years in NHANES 2005–2010, including children excluded from our analysis (n = 7,916; data not shown), sealant prevalence was 32.9% (SE = 1.0%). Sealant prevalence did not differ between children included and excluded from our study (Table 1). Furthermore, the sample used in this analysis (n = 7,090) was comparable to the total number of children in the combined NHANES database (n = 8,275; data not shown).

**Table 1:** Characteristics of Children Aged 6 to 19 Years Included in or Excluded From Study, NHANES 2005–2010

Variable	Included in Study (n = 7,090)		Excluded From Study (n = 1,185)		*P* Value^b^
	No.^a^	% (SE)	No.	% (SE)	
**Parental education**					
<High school diploma	2,133	19.2 (1.08)	329	26.4 (2.38)	<.001
High school diploma	1,687	23.8 (1.21)	222	29.9 (2.67)	
>High school diploma	3,270	57.0 (1.40)	295	43.7 (2.98)	
**Family’s income, % of the federal poverty level**					
<100	2,180	21.2 (1.10)	226	24.9 (3.04)	.22
100–200	1,916	22.4 (0.95)	167	19.7 (2.25)	
>200	2,994	56.4 (1.58)	235	55.4 (4.11)	
**Child’s age group, y**					
6–11	3,146	43.2 (0.95)	396	32.1 (1.83)	<.001
12–19	3,944	56.8 (0.95)	789	67.9 (1.83)	
**Child’s sex**					
Female	3,480	48.6 (0.80)	587	51.5 (1.76)	.15
Male	3,610	51.4 (0.80)	598	48.5 (1.76)	
**Child’s race/ethnicity**					
Mexican American	2,007	12.9 (1.34)	403	15.3 (1.74)	.009
Non-Hispanic black	1,936	14.3 (1.21)	318	16.0 (1.71)	
Other	979	12.2 (1.07)	192	16.7 (2.21)	
Non-Hispanic white	2,168	60.6 (2.13)	272	52.0 (3.14)	
**Health insurance**					
No insurance	1,086	11.4 (0.83)	224	15.0 (2.08)	.40
Medicaid/SCHIP	1,684	17.4 (1.07)	243	15.9 (2.03)	
Other government	885	9.3 (0.90)	139	9.1 (1.72)	
Private or military insurance	3,435	61.9 (1.70)	485	60.0 (2.94)	
**Regular source of care**					
No	688	7.5 (0.61)	186	12.3 (1.59)	.002
Yes (≥ 1 place)	6,402	92.5 (0.61)	996	87.7 (1.59)	
**Child’s general health**					
Fair to poor	490	4.9 (0.31)	110	7.3 (1.03)	<.001
Good	1,945	23.0 (0.69)	399	30.1 (1.57)	
Excellent to very good	4,655	72.1 (0.79)	675	62.8 (2.23)	
**Survey year**					
2005–2006	2,876	34.2 (1.95)	443	27.6 (2.96)	.08
2007–2008	2,047	32.5 (1.80)	379	38.3 (3.41)	
2009–2010	2,167	33.2 (1.77)	363	34.1 (3.17)	
**Sealant**					
No	5,056	66.8 (1.04)	611	69.5 (2.77)	.35
Yes	2,034	33.2 (1.04)	215	30.5 (2.77)	

Abbreviations: NHANES, National Health and Nutrition Examination Survey; SCHIP, State Children’s Health Insurance Program; SE, standard error.

a Sample sizes are unweighted; percentages estimated from weighted data.

b χ^2^ used to test for significance.

Sealant prevalence was associated with all independent variables (Table 2). Sealant prevalence increased with level of parental education and family income. Sealant prevalence was also higher among older children, girls, and non-Hispanic white children compared with younger children, boys, and non-Hispanic black and Mexican American children. Children with private health insurance and those with a usual source of care also had higher sealant prevalence than did children with Medicaid/SCHIP, other government insurance, or no insurance and children without a usual source of medical care. Sealant prevalence also was higher among children in excellent to very good health compared with those in good or fair/poor health — the indicator used for future need for dental care in this analysis. Finally, compared with previous NHANES cycles, sealant prevalence was higher in 2009–2010.

**Table 2:** Unadjusted Associations Between Prevalence of Sealants and Independent Variables Among Children Aged 6 to 19 Years, NHANES 2005–2010

Variable	With Sealants (n = 2,034)% (SE)	*P* Value^a^
**Parental education**		
<High school diploma	24.5 (1.58)	<.001
High school diploma	30.7 (1.78)	
>High school diploma	37.1 (1.18)	
**Family’s income, % of the federal poverty level**		
<100	25.7 (1.54)	<.001
100–200	28.1 (1.85)	
**>**200	38.0 (1.25)	
**Child’s age group, y**		
6–11	29.2 (1.27)	<.001
12–19	36.2 (1.34)	
**Child’s sex**		
Female	35.4 (1.34)	<.001
Male	31.1 (1.30)	
**Child’s race/ethnicity**		
Mexican American	28.5 (1.33)	<.001
Non-Hispanic black	22.3 (1.48)	
Other	34.1 (2.21)	
Non-Hispanic white	36.5 (1.56)	
**Health insurance**		
No insurance	23.1 (2.07)	<.001
Medicaid/SCHIP	27.2 (1.84)	
Other government	33.4 (2.55)	
Private or military insurance	36.6 (1.10)	
**Regular source of care**		
No	23.1 (3.00)	.04
Yes (≥ 1 place)	34.0 (1.03)	
**Child’s general health**		
Fair to poor	27.3 (2.47)	<.001
Good	27.1 (1.65)	
Excellent to very good	35.5 (1.12)	
**NHANES survey year**		
2005–2006	32.3 (2.27)	.001
2007–2008	26.0 (1.04)	
2009–2010	41.0 (1.92)	

Abbreviations: NHANES, National Health and Nutrition Examination Survey; SCHIP, State Children’s Health Insurance Program; SE, standard error.

a χ^2^ used to test for significance.

Before controlling for potential covariates, we found that sealant prevalence differed only by parental education among the middle- and high-income groups (*P* values for lowest income group always exceeded .80; data not shown). Among the middle-income group (100%–200% of the FPL), the differences in sealant prevalence among children of parents who had more than a high school diploma (prevalence, 30%) or a high-school diploma (28%) compared with less than a high school diploma (22%) were 8 percentage points (*P* = .007) and 6 percentage points (*P* = .04), respectively. Among the highest income group (>200% of the FPL), the differences in sealant prevalence between children of parents who had more than a high school diploma (40%) or graduated high school (35%) compared with parents who were not high school graduates (25%) were 15 percentage points (*P* < .001) and 10 percentage points (*P* = .04), respectively.

### Logistic regression results

**Figure 2:**
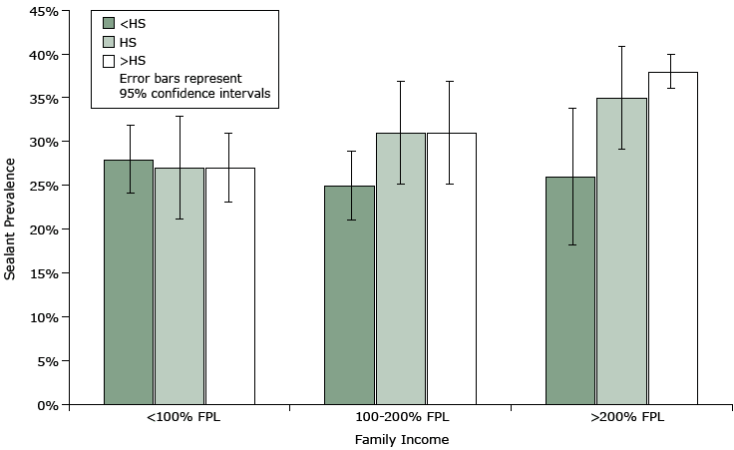
Adjusted sealant prevalence by education and family income, National Health and Nutrition Examination Survey (NHANES), 2005–2010. Abbreviations: FPL, federal poverty level; HS, high school graduate.

After controlling for potential covariates, the association between sealant prevalence and education was still modified by income (Table 3). The odds of having a sealant among children of parents who were not high school graduates versus parents who had more than a high school education were significant among children from families with incomes at 100% of the FPL or greater. Among families with the highest income (>200% of the FPL), sealant prevalence estimated from our regression model was approximately 12 percentage points higher (38.2% vs 25.7%) for children of parents with more than a high school diploma (Figure 2) compared with those who did not graduate high school.

**Table 3:** Odds Ratios (ORs) With 95% Confidence Intervals (CIs) From Multiple Logistic Regression Model^a^ of Children Aged 6 to 19 Years With at Least 1 Sealant, NHANES 2005–2010

Variable	OR (95% CI)	*P* Value^b^
**Combined effect of income and education**		
**<100% FPL**		
<High school diploma vs high school diploma	1.03 (0.75–1.42)	.85
<High school diploma vs >high school diploma	1.07 (0.83–1.37)	.62
High school diploma vs >high school diploma	1.03 (0.70–1.52)	.86
**100%–200% FPL**		
<High school diploma vs high school diploma	0.72 (0.52–1.01)	.05
<High school diploma vs >high school diploma	0.73 (0.53–1.00)	.05
High school diploma vs >high school diploma	1.01 (0.73–1.39)	.96
**>200% FPL**		
<High school diploma vs high school diploma	0.64 (0.39–1.07)	.09
<High school diploma vs >high school diploma	0.55 (0.36–0.83)	.005
High school diploma vs >high school diploma	0.85 (0.64–1.12)	.24
**Child’s age group**		
6–11	0.67 (0.58–0.79)	<.001
12–19	1.0 [Reference]	
**Child’s sex**		
Female	1.24 (1.06–1.45)	.008
Male	1.0 [Reference]	
**Child’s race/ethnicity**		
Mexican American	0.98 (0.78–1.22)	.85
Non-Hispanic black	0.59 (0.48–0.72)	<.001
Other	1.01 (0.79–1.30)	.91
Non-Hispanic white	1.0 [Reference]	
**Health insurance**		
No insurance	0.72 (0.58–0.90)	.005
Medicaid/SCHIP	1.01 (0.80–1.29)	.90
Other government	1.26 (0.98–1.62)	.08
Private or military insurance	1.0 [Reference]	
**Regular source of care**		
No	.70 (0.53–0.93)	.01
Yes (≥one place)	1.0 [Reference]	
**Child’s general health**		
Fair to poor	0.87 (0.66–1.16)	<.001
Good	0.74 (0.63–0.87)	.33
Excellent to very good	1.0 [Reference]	
**Survey year**		
2005–2006	0.67 (0.50–0.88)	.006
2007–2008	0.50 (0.41–0.61)	<.001
2009–2010	1.0 [Reference]	

Abbreviations: OR, odds ratio; FPL, federal poverty level.

a Hosmer-Lemeshow Satterthwaite, *P* = .55.

b Wald *F*-test used to test for significance of combined effect of income and education and Wald *t* test used for other variables.

The findings from our logistic regression were similar to those from the bivariate (unadjusted) analysis. Younger children, boys, and non-Hispanic black children were less likely to have a sealant than were older children, girls, and non-Hispanic white children. Furthermore, uninsured children and those without a regular source of care had lower odds of having a sealant than did privately insured children and children with a regular source of care. Finally, the odds of having a sealant were still higher among children reporting better general health and those in the last cycle of NHANES (Table 3).

Unlike the bivariate analysis, however, the multivariate analysis did not find a difference in the odds of having versus not having at least 1 sealant between Mexican American children and non-Hispanic white children (OR, 0.99; 95% CI, 0.80–1.24). Similarly, the odds of having sealants did not differ between Medicaid and privately insured children (OR, 1.02; 95% CI, 0.81–1.29).

## Discussion

This study used Andersen and Aday’s PEN model of health care utilization (17) to examine whether the association between sealant prevalence in children and the predisposing factor, parental education, varied by the enabling factor of family income, controlling for other predisposing, enabling, and need variables. Andersen’s model has been expanded since its initial conception to include additional variables (18) but, as suggested by the model’s authors, we used the original model because of data availability and its suitability to our research question (17). We found that, although sealant prevalence did not vary by level of parental education among children from low-income families, having a parent who was educated beyond high school (vs a parent who did not graduate high school) was associated with an almost 50% increase in sealant prevalence among children from high-income families (26% among <high school diploma vs 38% among >high school diploma).

To the extent that education and income are good measures of predisposing and enabling variables, respectively, our findings suggest that parents predisposed to having their child receive sealants require sufficient enabling resources or that parents with sufficient enabling resources must be predisposed to having their child receive sealants. We classified education as a predisposing factor because of its strong association with health literacy (12,13), which in turn predicts higher sealant prevalence (10). This classification is also consistent with Andersen and Aday’s identification of education as a predisposing factor in their PEN model (17).

Although we had 3 measures of enabling resources (income, health insurance, and usual source of health care), we used income as the primary proxy for enabling resources. We did so because the other 2 items measured enabling resources for medical as opposed to dental care. Analyses conducted during the 2005 to 2010 timeframe of this study suggest that approximately one-fifth of children with health insurance do not have dental insurance — approximately 94% of US children had health insurance (23) while only approximately 75% had dental insurance (24). In addition, health insurance may be more enabling for health care than dental insurance is for dental care — dental insurance has higher copays and lower annual limits than medical insurance and, as a result, approximately 40% of dental expenditures are paid out-of-pocket (25).

The finding that increased education was not associated with increased sealant prevalence among children from low-income families is noteworthy because these children are eligible to receive Medicaid dental benefits in all states (26). Medicaid dental coverage, however, may be less enabling than private dental insurance. Dentists may be less likely to participate in Medicaid for reasons such as lower reimbursement rates. In 2013, for example, the average Medicaid fee-for-service reimbursement for children’s dental services was less than half (48.8%) that of commercial dental insurance charges (26).

Our findings deviated from the PEN model in that the variable we included to reflect future need for care (poor perceived child’s health status) was associated with lower sealant prevalence. These results are similar to those from another study of factors influencing receipt of preventive medical and dental care where children with self-reported poor health status were less likely to have received preventive pediatric health and dental care (27). Poor general health may not only indicate higher caries risk but also poorer access to care due to limitations in mobility or financial resources. Children in poor health may use more treatment or acute care services at the expense of needed preventive services (27).

Our study has limitations. Because this analysis used cross-sectional data, our findings can be interpreted only as an association rather than a causal factor of children’s having sealants. In addition, family income, parental education, insurance, and health status were self-reported with no objective measures to confirm validity. A final limitation was the change in the NHANES examiner type from health technologists to dental hygienists between 2005–2008 and 2009–2010. However, the clinical assessment criteria did not change nor did the reference examiner change and any potential differences because of the change in examiner type should be partially captured by the survey year variable.

Our findings suggest that the impact of higher parental education on a child’s having sealants is greater in the presence of higher family income and that the impact of higher family income on a child’s having sealants is greater in the presence of higher parental education. Interpreting our findings in the context of oral health care for children in the United States suggests that sealant prevalence could increase. Recent health care reforms will probably enable more families to obtain preventive dental services (including sealants) for their children through increased access to dental insurance and increased supply of dental providers (4). Andersen and Aday argued that addressing enabling variables would be the most effective strategy to achieve equitable access to health care, as these resources could be altered with changes in government policy (17). Predisposing factors such as education, however, are considered less mutable in the short run. If education is indeed capturing the influence of health literacy, then it may be possible to alter a parent’s predisposition toward sealants with health literacy campaigns. The renewed focus on the importance of oral health literacy could in a short time result in more families being aware of the importance of good oral health and the preventive benefits of dental sealants. Health and oral health literacy are a focus of interest at the national level, as demonstrated in the recommendations from a recent Institute of Medicine workshop (28), objectives in Healthy People 2020 (7), and the Health and Human Services National Action Plan to Improve Health Literacy, which includes an oral health component (29).

## Figures and Tables

**Table:** 

**1. The activity supported the learning objectives.**				
**Strongly Disagree**				**Strongly Agree**
1	2	3	4	5
**2. The material was organized clearly for learning to occur.**				
**Strongly Disagree**				**Strongly Agree**
1	2	3	4	5
**3. The content learned from this activity will impact my practice.**				
**Strongly Disagree**				**Strongly Agree**
1	2	3	4	5
**4. The activity was presented objectively and free of commercial bias.**				
**Strongly Disagree**				**Strongly Agree**
1	2	3	4	5

## Post-Test Information

To obtain credit, you should first read the journal article. After reading the article, you should be able to answer the following, related, multiple-choice questions. To complete the questions (with a minimum 75% passing score) and earn continuing medical education (CME) credit, please go to http://www.medscape.org/journal/pcd. Credit cannot be obtained for tests completed on paper, although you may use the worksheet below to keep a record of your answers. You must be a registered user on Medscape.org. If you are not registered on Medscape.org, please click on the "Register" link on the right hand side of the website to register. Only one answer is correct for each question. Once you successfully answer all post-test questions you will be able to view and/or print your certificate. For questions regarding the content of this activity, contact the accredited provider, CME@medscape.net. For technical assistance, contact CME@webmd.net. American Medical Association's Physician's Recognition Award (AMA PRA) credits are accepted in the US as evidence of participation in CME activities. For further information on this award, please refer to http://www.ama-assn.org/ama/pub/about-ama/awards/ama-physicians-recognition-award.page. The AMA has determined that physicians not licensed in the US who participate in this CME activity are eligible for ***AMA PRA Category 1 Credits*** ™. Through agreements that the AMA has made with agencies in some countries, AMA PRA credit may be acceptable as evidence of participation in CME activities. If you are not licensed in the US, please complete the questions online, print the AMA PRA CME credit certificate and present it to your national medical association for review.

## Post-Test Questions

### Article Title: Exploring the Effect of Family Income on the Relationship Between Parental Education and Sealant Prevalence

**CME Questions**

1. You are seeing a 10-year-old girl with a history of dental caries, and you consider applying a sealant for this patient. What should you consider regarding the epidemiology of caries and the effectiveness of dental sealants?
  - Dental caries affect all children equally, regardless of family income
  - Sealants may reduce the incidence of caries by more than 80% after 2 years
  - Sealants are ineffective at preventing the progression of early caries
  - Most children living in poverty have received dental sealants
2. You discuss the application of a dental sealant with the girl's parents. According to bivariate analysis in the current study by Al Agili and colleagues, what were the effects of parental income and education on the use of sealants?
  - Higher income was associated with a lower rate of sealant use; higher educational status was associated with a lower rate of sealant use
  - Lower income was associated with a lower rate of sealant use; higher educational status was associated with a lower rate of sealant use
  - Lower income was associated with a higher rate of sealant use; lower educational status was associated with a lower rate of sealant use
  - Lower income was associated with a lower rate of sealant use; lower educational status was associated with a lower rate of sealant use
3. What more did the current study demonstrate regarding the relationship between parental income, educational attainment, and the application of dental sealants?
  - The effect of higher educational attainment promoted higher sealant use primarily among lower-income families
  - The effect of higher educational attainment promoted higher sealant use primarily among higher-income families
  - Only educational attainment beyond a collegiate degree was associated with lower sealant levels among higher-income families
  - Higher educational attainment promoted higher sealant use regardless of family income level
4. Which of the following other variables was a significant risk factor for failure to receive a dental sealant in the current study?
  - Non-Hispanic black race
  - Mexican American ethnicity
  - Medicaid insurance
  - Younger age

**Evaluation**
